# Conceptualizing the effective mechanisms of a social needs case management program shown to reduce hospital use: a qualitative study

**DOI:** 10.1186/s12913-022-08979-z

**Published:** 2022-12-26

**Authors:** Mark D. Fleming, Nadia Safaeinili, Margae Knox, Elizabeth Hernandez, Emily E. Esteban, Urmimala Sarkar, Amanda L. Brewster

**Affiliations:** 1 University of California, Berkeley, School of Public Health—Berkeley, California, USA; 2 Contra Costa Health Services, Contra Costa County—Concord, California, USA; 3 Department of Medicine—San Francisco, University of California, San Francisco, California, USA

**Keywords:** Case management, Social needs, Social determinants of health, High-risk complex patients, Care coordination

## Abstract

**Background:**

Social needs case management programs are a strategy to coordinate social and medical care for high-risk patients. Despite widespread interest in social needs case management, not all interventions have shown effectiveness. A lack of evidence about the mechanisms through which these complex interventions benefit patients inhibits effective translation to new settings. The CommunityConnect social needs case management program in Contra Costa County, California recently demonstrated an ability to reduce inpatient hospital admissions by 11% in a randomized study. We sought to characterize the mechanisms through which the Community Connect social needs case management program was effective in helping patients access needed medical and social services and avoid hospitalization. An in-depth understanding of how this intervention worked can support effective replication elsewhere.

**Methods:**

Using a case study design, we conducted semi-structured, qualitative interviews with case managers (*n* = 30) and patients enrolled in social needs case management (*n* = 31), along with field observations of patient visits (n = 31). Two researchers coded all interview transcripts and observation fieldnotes. Analysis focused on program elements identified by patients and staff as important to effectiveness.

**Results:**

Our analyses uncovered three primary mechanisms through which case management impacted patient access to needed medical and social services: [1] Psychosocial work, defined as interpersonal and emotional support provided through the case manager-patient relationship, [2] System mediation work to navigate systems, coordinate resources, and communicate information and [3] Addressing social needs, or working to directly mitigate the impact of social conditions on patient health.

**Conclusions:**

These findings highlight that the system mediation tasks which are the focus of many social needs assistance interventions offered by health care systems may be necessary but insufficient. Psychosocial support and direct assistance with social needs, enabled by a relationship-focused program, may also be necessary for effectiveness.

## Introduction

Although health care systems are increasingly exploring interventions to assist patients with social needs [1, 2], a lack of evidence about the mechanisms by which these complex interventions benefit patients challenges translation across settings and populations. In social needs case management [3–5], a professional works collaboratively with a patient to identify, coordinate, and advocate for services, which may span the medical, behavioral health, and social care systems [6]. Social needs assistance is often targeted to individuals with high levels of prior utilization, with the hope that case management can improve health and reduce use of hospital services. Interventions can vary from brief screening and referral to social services to multi-year case management relationships.

Our present study investigates the mechanisms of effectiveness of the CommunityConnect social needs case management intervention, recently shown to reduce inpatient hospitalizations by 11% in a randomized study [7]. Prior studies of social needs assistance in health care have shown highly varied impacts on patient outcomes, ranging from no impact to some reduction in health care utilization [5, 8–10]. As complex interventions embedded in distinct health care systems, components of social needs assistance programs likely need to vary across settings. Complex interventions have multiple independent and interdependent components, which can make it difficult to specify the active ingredient and to replicate the results of a complex intervention in new settings [11]. Studies to reveal processes and mechanisms of action have been identified as crucial for evaluating complex interventions [12]. Therefore, there is an important need for research to illuminate the mechanisms through which effective social needs case management interventions achieve desired effects.

Previous evaluations of effective social needs case management have not included in-depth studies of key mechanisms of impact [13], which can differ from intervention designers’ expectations and protocols [14]. Social needs assistance may influence patients’ health through multiple mechanisms such as by improving the tailoring of health care, reducing patient stress and provider burnout [5], increasing access to medications [15], and trust building and engagement [16]. While previous literature has called out the importance of addressing social needs for health [17], few studies explicate the tasks or processes to do so.

Therefore, our study aimed to generate an in-depth understanding of how the CommunityConnect intervention was effective in helping patients access needed medical and social services and avoid hospitalization. This information can elucidate priorities for the design and implementation of social needs case management programs elsewhere.

## Methods

### Study design

We used an in-depth, qualitative case study design to investigate the mechanisms by which case management assisted patients, as qualitative methods are ideal for illuminating complex phenomena that depend on context and interpersonal relationships [18].

### Setting and intervention

In 2017, Contra Costa Health Services in California implemented the CommunityConnect social needs case management program as part of California’s Whole Person Care Medicaid 1115 waiver program. CommunityConnect employed about 70 in-person case managers with fulltime caseloads of about 90 patients each, serving up to 6000 patients total at a time. Patients were administratively enrolled into the program based on a risk model predicting avoidable hospitalization and ED use. Case managers aimed to meet patients at least once monthly for 1 year, although frequency of contact varied based on patient needs. About 40% of enrollees engaged in services, with an average of 17 contacts with their case manager. An evaluation using a randomized encouragement design demonstrated the program reduced acute care use in the intervention group [7].

Case managers offered similar services to all patients, including initial social needs screening, psychosocial support and motivational interviewing, and coordinating medical and social services. Case managers came from multiple professional disciplines and were organized into multidisciplinary teams to conduct case conferencing. In addition to connecting patients to health services and community-based resources, CommunityConnect directly provided mobile phones, transportation vouchers, and housing vouchers to patients with needs for these resources.

### Participants and data collection

Case managers were selected for recruitment by purposive sampling to represent diverse disciplines, gender, and language (to ensure inclusion of case managers who primarily spoke with patients in languages other than English). Case managers were included if they had at least 1 year of experience. Of the approximately 70 case managers, 33 case managers were invited for interviews and observations by email; 31 completed in-person interviews in the CommunityConnect offices (Table 1). Patients were included in the study if they were enrolled in CommunityConnect, which included adults aged 18 or older residing in Contra Costa County with full-scope Medicaid who were not in another case management program. There were no explicit exclusion criteria for participation. Patients were identified and asked to participate by their case managers, resulting in 31 patient interviews and field observations of case manager visits, both of which took place largely in patients’ homes. For observations, we recruited the same case managers and patients who participated in interviews. Observations took 45–60 minutes and patient interviews 15–30 minutes. No participant declined observations. We used non-participant observation and documented interactions with detailed fieldnotes.

**Table 1:** Interview Sample by Case Manager Discipline

	# Case managers	# Interviewed
Public Health Nurse	28	9
Substance Use Counselor	12	5
Community Health Worker Specialist	9	2
Social Worker	8	6
Mental Health Clinical Specialist	7	4
Homeless Services Specialist	6	4
Total	**70**	**30**

CommunityConnect evaluation staff members (AS, EH, CT, EE, GQ, JS) conducted interviews and observations with case managers and patients. All data collection took place between March 2019 and November 2019. Interviews were audio recorded with participant permission and transcribed verbatim. Spanish language interviews and observations were conducted by Spanish speaking researchers. Spanish language transcripts were translated to English for analysis. All transcripts and fieldnotes were uploaded to Nvivo12 for analysis.

### Data analysis

The research team took an integrated approach to codebook development [19]. To build on existing knowledge of social needs case management, we first derived deductive codes from specific program services (e.g. medical, substance use, mental health) and patient health and social needs (e.g. housing, food and nutrition, transportation, finance and public benefits). We then integrated inductive codes capturing a range of emergent themes on topics related to outreach and engagement, rapport and trust building, system navigation, and health education. Five researchers coded interview transcripts; all data were coded by two researchers to ensure consistency of coding.

To identify potential mechanisms of effectiveness, we first defined the primary components of the intervention by analyzing data coded for case manager activities. Then, using the constant comparative method [20], we compared the component analysis to data capturing patient experiences and perspectives and identified key mechanisms though which case management impacted patients. We defined mechanisms as the activities and process that case managers and patients reported as important for benefiting patients, and thus did not aim to characterize the full range of case manager activities. Rather than inventory the range of tasks that case managers perform, as other studies have done [21, 22], we focused on what case managers and patients reported to be the most important mechanisms of case management.

## Results

Public health nurses were the most represented case manager discipline, comprising nearly one in three case managers interviewed (9 out of 30). Other disciplines interviewed included social workers (6 out of 30), substance use counselors (5 out of 30), mental health clinical specialists (4 out of 30), homeless services specialists (4 out of 30), and community health worker specialists (2 out of 30) [Table 1]. Among patient participants, about half (15 out of 31) were age 60 or above. Patient participants were largely White/Caucasian (13 out of 31), although Black/African American participants and Hispanic/Latino participants each represented 16% of interviewees. Most interviewees had multiple behavioral, mental, and physical conditions including alcohol and other drug dependence (14 out of 31), depressive disorder (21 out of 31) and hypertension (22 out of 31) [Table 2].

**Table 2:** Sample of patient participants

Age		
20 to < 40	4	13%
40 to < 60	12	39%
60 and above	15	48%
Race/Ethnicity		
White/Caucasian	13	42%
Black/African American	5	16%
Hispanic/Latino	5	16%
Asian	2	6%
Other	6	19%
Language		
English	29	94%
Spanish	2	6%
Selected Conditions (patient may have multiple)		
Alcohol and Other Drug Dependence	14	45%
Chronic Obstructive Pulmonary Disease	12	39%
Chronic Pain	21	68%
Depressive Disorder	21	68%
Hypertension	22	71%

**Fig. 1:**
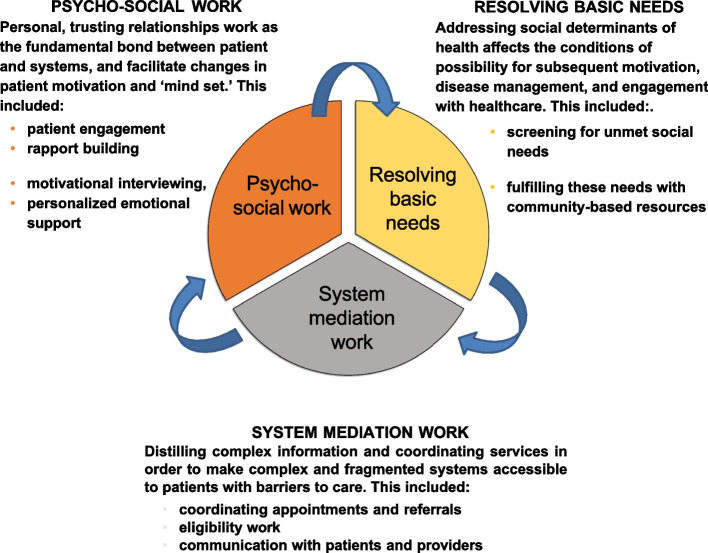
Social needs case management programs impact patient outcomes through three primary mechanisms

Our analyses uncovered three key mechanisms through which case management impacted patients: [1] Psychosocial work, defined as interpersonal and emotional support provided through the case manager-patient relationship, [2] System mediation work to navigate systems, coordinate resources, and communicate information and [3] Addressing social needs, or working to directly mitigate the impact of social conditions on patient health. While these themes represent qualitatively distinct mechanisms, we found that they were closely intertwined and mutually reinforcing (Fig. 1).

### Psychosocial work

A consistent theme shared by interviewees was that the nature and quality of the relationship between case manager and patient was foundational to the effectiveness of the program. Personal, trusting relationships worked as the basis for initial acceptance of services, changes in patient motivation and “mind set,” and maintaining engagement over time and in the face of setbacks. Case managers devoted significant time to patient outreach and engagement, rapport building, and personalized emotional support. Emotional support included helping patients manage mental health symptoms. In the participants’ view, case management was effective when the case manager was reliable, empathetic, and non-judgmental, and demonstrated a sense of “care.”

When patients were first introduced to the program, they often had a skeptical attitude towards service providers. Developing a trusting, personal relationship was key to overcoming initial distrust:

Case Manager 24: A lot of our population has been let down by professionals in the human services field. So, we come trying to provide or instill hope and…we have to spend a lot of our time just trying to garner a relationship with a client. No one’s really going to be receptive to case management unless some kind of trust or reputation is established first. So, I think, trust, building trust, getting someone to a point where they trust your opinion, they’re comfortable with moving in the direction that you point them to.

Participants reported that a sense of “care” was important for establishing a trusting relationship and engaging patients in services:

Case Manager 33: A lot of my clients say “You know I feel like you really care about me. You help me out a lot. I am really thankful that you actually care.” It’s a big plus in helping that, some clients don’t trust anybody or kind of feel like we’re authority…because we work for the county. But knowing that they have someone that they can trust is a big help in their recovery.

Patient 11: My CommunityConnect worker, I latched on to her. Hey, somebody on my side that cares and wants to help me get my stuff together.

Case managers emphasized that listening empathetically and non-judgmentally was a key skill for effective case management and, correspondingly, patients reported that feeling heard and having their individual perspectives acknowledged by case managers was something they valued most about the program. Case Manager19 said that an effective case management practice was, “To listen to them and to ask them what they want and what they want to do, what they want to do with their lives, how we can help them to get there.” Patients often attributed their engagement in the program to the sense of feeling listened to and cared for.

Patient 09: I have never come across a city worker or a county worker who’s been so compassionate, never in my life. And because of that, I think I’ve stuck with the program more because I don’t feel as a number. She made me feel as a person.

Case managers and patients also reported that personalized, emotional support improved patient psychological wellbeing and helped manage mental health symptoms such as anxiety and depression.

Patient 11: I can talk to them [the case managers] and I feel comfortable talking to them about things…Sometimes its personal issues that bother me [that] I’ve been able to get off my chest because I don’t have a psychiatrist to talk to or an analyst to talk to about personal issues that bother me. My CommunityConnect worker does all that you know.

Patient 01: I have anxiety, I suffer really bad panic attacks, but I always know, in the end, I feel good [after meetings with Case Manager4]. Case Manager4 coming here [for home visits] really helps me out a lot versus me having to go out sometimes when my anxiety goes high.

The case manager being reliable and consistently available was important for maintaining trust and engagement over time.

Case Manager 26: Um just showing up and not giving up on a continual basis. Regardless of their forward movement and their back movement, right cause that happens a lot.

Patient 28: The days she tells me she’s going to come, she comes. And I feel calm with her because she helps me.

### System mediation work

We defined system mediation work as the broad set of activities to coordinate services and to make systems accessible to patients. Case managers spent a significant proportion of their time coordinating information and services within and across medical, behavioral health, and social service organizations. While this work required an established foundation of a personal, supportive relationship (psychosocial work), system mediation encompasses distinct activities focused on helping guide patients through fragmented services landscapes, overcoming logistical barriers to care, and communicating relevant information to patients and other providers.

A key function of the case management program was to create connections between patients and services, which required overcoming barriers associated with system fragmentation. As Case Manager 23 described it, this work entailed “Being that person in the middle and advocating for them to access certain services.” Case Manager 13 stated:

I will describe it as being sort of like a bridge between the need our clients have to the resource that they need. And being the bridge that gets them the help and the resource, and it covers that extra space, or emptiness, or problem that they have.

Case managers helped patients access systems by serving as a “go to” person, or single point of contact or entryway into systems.

Case Manager 29: It’s just like you call on the phone and they say, “Push 1 if you want to leave a message, or push 2.” You know you keep on saying what you want and you never get a live person…a live person never comes on. So here we are. We are live persons. We’re live, we’re here to help you. A person, face to face, trying to help you make it through the system.

Patient 30: I have a person that I can go to now…It’s just sometimes when something comes up, comes in the mail, instead of freaking out I’ll text her and she text’s me back.

Study participants reported that overly complex systems and lack of knowledge about how to access and navigate systems contributed to patient health crises and hospitalizations. Case management worked to overcome these barriers by offering direct assistance in the navigation process.

Case Manager 02: One of the big things honestly is the ability to navigate systems…The ability to read documents. The ability to operate technology devices like phones and computers. The ability to figure out bus schedules. So, to already have certain skill sets in place, some of my clients don’t have those life skills and aren’t set up.

Patient 28: She helps me fill out forms that I don’t understand and all that. She’s even sent them for me and all that.

Patient 30: It really helps you go through the pitfalls of getting things, paperwork, to be done. And just really getting your foot into the door about programs that I did not know about. The rideshare, Contra Costa transportation, I never knew about that…I don’t know any of this lingo or mumbo jumbo I mean I don’t know or say the right thing you know. She does.

In addition to directly assisting patients with system mediation, case managers used interactions with patients as opportunities to provide coaching and motivation for patients to access and use resources. Case managers described a progression that began with providing direct assistance and, over time, coaching some patients to navigate systems on their own.

Patient 03 described experiencing anxiety and confusion when communicating when service providers and said, “When we do that together it’s like more of a push, you know. I kind of need the push sometimes, often I’ll be scared or busy to take things myself and she’ll remind me, and we will just do it together.” Case Manager 03 stated:

My goal is to help them attain their goals. To help them be more independent within the system as well. Teaching and providing education in instruction on how to schedule their own appointments. Or how to access services or benefits for themselves. Often times our patients aren’t clear on how they system works. Maybe what they can access or don’t have access to. Education and skill building is a big part in what I do.

Mediation work included ongoing health education, which involved distilling and communicating health care information. Case managers helped translate diagnoses and recommendations regarding medications and lifestyle changes into terms that were easier to for patients understand.

Case Manager 26: making sure they’re…educated about their medications, making sure they understand what health issues they do have and how to manage it.

In addition to working directly with patients, case managers reported that a crucial aspect of their work was communicating with a variety of service providers and organizations to coordinate efforts and services, facilitate referrals, and to advocate for patients.

Case Manager 28: Networking is very important, not only with your patients but with people in the community and engaging other community resources.

Case Manager 02: I communicate with a variety of providers. I communicate with psychiatrists. I communicate with care coordinators, primary care providers. If there in an assisted living facility I communicate with the providers there. I communicate with providers at paratransit. I also communicate with community-based organizations…I then communicate with my Mental Health team. As well as my disciplinary team. I communicate with clerks here about transportation.

### Addressing social needs

Study participants reported that the capacity to address social needs was important for the effectiveness of the program. In CommunityConnect, staff were trained to use a social needs using screening tool with open-ended questions and to match needs with available community-based resources. Further, program organizational supports for addressing social needs included a database of available social services resources, formal resource sharing agreements between health care and social services organizations, and established cross-sector referral pathways.

Case managers in our study identified this capacity to address needs like housing, food, transportation, and income as central to how the program benefits patients and reduces hospital use.

Case Manager 08: I feel like the program has the right idea. I think that if you’re able to get people’s social needs met, their quality of life is going to be better. I mean I have been at the hospital before. People are just there [because] they want to eat. They want a place to sleep. They want to get out of the cold.

Our findings also revealed that case managers used a distinctive social determinants of health conceptual framework to orient their work activities. Case managers understood social conditions as “root causes” of health problems and hospital use. They sought to identify underlying social drivers and mitigate social needs as a first step towards supporting disease management.

Case Manager 10: Social determinants can significantly affect their ability to get health care, to access health care, maintain health care. And eating healthy and transportation, or, housing, so that’s how I explain [the program]…I think the world is beginning to realize people are whole people and you can’t just treat the symptom. You have to look at root cause and you have to deal with that. Otherwise, the cost savings isn’t there because 2 months they’ll be back here for the same thing in the hospital.

Case managers met patients at their homes or in a community location to perform the initial social needs screening. The screening tool consisted of 42 questions covering medical, dental, vision, behavioral health, safety, housing, finances, food security, transportation, social support, and legal needs. Case managers performed social needs screening as an in-depth conversation, sometimes split across multiple conversations, and used motivational interviewing to explore social needs, medical needs, priorities and goals.

Case Manager 25: I’m collecting a lot of information, a lot of dialogue about their history. How did they wind up if they’re homeless? Or if there is a loss of income, how did they get there?

While screenings often turned up a range of needs, case managers focused their work on patient identified priorities.

Case Manager 23: Each client needs something different. I may be working on a housing goal with one client and may be working with substance abuse with another client. It may be linking to a job opportunity for another client…So it just depends on where that client is and what goals that they specify that they need to work on in order to get their lives better quality.

Case managers also reported that patient priorities were sometimes not aligned with identified needs. In the case managers’ perspective, some patients were not “ready” to address their most pressing needs.

Case Manager 21: First thing is, they are the boss. They live their lives and I can’t make them do one thing or another... So, they have to be willing. We kind of negotiate. I try to basically explain the rationale behind it and why this is a priority, so they understand. If your blood sugar is consistently 500 or 600, you know, if we try to deal with something less important then it won’t help you because this will destroy your health… at the end you can’t make people do anything… So, they have to want that and be willing to do their part.

By assessing patients’ medical and social needs in an integrated fashion and through the lens of social determinants of health, case managers took a “whole person” approach. Case Manager 11 described their approach: “Work collaboratively to look at how psychosocial stressors are related to poverty and the client’s outcome; whole picture; seeing social needs as included in health outcomes.”

Case Manager 14: The person’s mental health and their income and their medical care are not three, like the same person chopped into three. It’s like all part of the same person.

Case managers and patients agreed that the programs’ capacity to address social needs supported subsequent engagement with health care and disease management:

Case Manager 05: Not being homeless, that’s the main thing they are concerned about. That and having food. This is why we give the food pantry resources to them so quickly. Or the food stamps. If they don’t have anywhere to go and they are hungry, they don’t really care about needing an eye appointment. Or anything else to be honest.

Case Manager 09: …a lot of it is working on social needs and building rapport so then we can get into the medical needs, the dental and the mental health and substance use.

Patient 22: [Explaining their motivation for engaging with their case manager] In all honesty in the beginning I was looking for the phone…and I needed the tools, the clothes and Case Manager 33 was on it.

By understanding social determinants as the conditions that make it possible to engage with routine medical care, case managers also took a destigmatizing approach to poor adherence, understanding it to be caused by unmet social needs.

The availability of community-based services was important for the effectiveness of the intervention. The program compiled a database of available services and developed a smartphone app for case managers and patients to identify appropriate services.

Case Manager 10: I’ve already seen a lot of my patients be empowered by it [the database and app], to the point where I talk to them now and they’re like, Oh I did this, I did that. Great! Excellent! Alright! And all I did was give them a few resources. A lot of times it’s like why don’t the general public know about all these resources.

At the same time, the program’s dependence on community-based resources limited what the case managers could offer, sometimes leading to the “road to nowhere problem” [23] when screening identified a social need but referral to a social resource was not successful.

In sum, case managers used the social determinants of health framework to prioritize and sequence their psychosocial and system mediation work with patient. We identified addressing social needs as a discrete mechanism because it involved a distinctive way of conceptualizing the causes of health problems (as outcomes of social drivers), integrating social and medical concerns, and crossing the boundaries between health care and social services.

## Discussion

Our analysis identified three distinct mechanisms that were identified by case managers and patients as important for impacting patients: psychosocial work, system mediation work, and addressing social needs [21, 22]. These findings support prior assumptions that social needs assistance interventions can benefit patients by helping them access social services [5, 8, 16], but also suggest that the effectiveness of this type of intervention depends on the interplay of multiple mechanisms of action.

Trusting relationships between patient and case manager have been found to help patients feel more secure and empowered to make decisions and take actions to support their health [16, 24]. Patient trust may also be an important indicator of success in case management programs [24]. Additionally, we found that a positive relationship with a case manager may help to mitigate the impacts of mental health challenges. Patients with high risk of acute care utilization are known to have elevated rates of mental health challenges, higher stress, and lower social support than those with less acute care use [25, 26]. Interventions targeting this population may consider equipping case managers with skills and resources for providing mental health counseling and support.

Many of the system mediation activities we identified fit within standard definitions of patient navigation or care coordination and include filling out paperwork, demonstrating eligibility requirements, and facilitating referrals, appointments, and movement across the continuum of care [27–29]. However, we found that case managers engaged in a broader set of activities that included distilling and communicating complex medical and social information, serving as a communicative link among networks of service providers and collaborating organizations, and using the EHR to document and communicate directly with medical and social services providers. Further, in helping patients navigate systems, case managers worked to coach and motivate patients to navigate systems themselves.

We found that addressing social needs is a distinctive activity involving social risk screening, integrating patients’ health care and social services priorities, and making linkages across the traditionally siloed health care and social services organizations [5, 30]. Prior research has suggested possible mechanisms through which social needs programs benefit patients, including by resolving unmet social needs, improving health care quality, reducing patient and provider stress, and improving access to medications and clinical appointments [5, 15]. In addition to these mechanisms, we found that addressing social needs involved conceptualizing health issues and health care use as effects of upstream social drivers. This lens informed case manager’s prioritization and sequencing of tasks. Without first addressing social needs, many patients could not be expected to engage in health care. Both case managers and patients reported that addressing unmet social needs led to subsequent engagement in health care services.

Our study is unique in its identification of multiple, synergistic mechanisms that contributed to positive outcomes for a social needs case management program with demonstrated effectiveness. Our results should be interpreted in light of several limitations. First, the data are derived from a single program, targeting a population with Medicaid insurance and moderately high risk of acute care utilization. It is possible that some of the mechanisms we identified would be less salient in other settings. CommunityConnect was relatively unique in that it enrolled patients using a risk model predicting avoidable hospital use. By contrast, a systematic review reported that most case management programs start at hospital discharge and provide transitional services [31]. Second, our study was conducted in collaboration with the implementing health system in which case managers worked, which might have shaped what case managers shared in interviews. Case managers shared a range of positive and negative experiences in interviews, however, suggesting that many case managers felt able to share candid observations. Third, case managers recruited patients for study participation and they may have selected more engaged or positive patients. This could mean that our results are less representative of patients for whom the intervention was effective but had a less engaged relationship with their case manager.

## Conclusions

Overall, this study helps to conceptualize how social needs case management may be achieving its effects (Fig. 1). Social needs intervention programs are often designed with only one or two of these mechanisms. For example, some programs use social needs screening, and only minimal support with referrals and no psychosocial support [5]. Our results suggest that more detailed characterization of these interventions is needed to understand which programs are comparable, and how they influence patients’ health and well-being. Further, the identification of program mechanisms provides a framework to describe implementation fidelity and support cross-program evaluations. Future research should consider the minimum mechanisms necessary to achieve desired outcomes for particular populations and settings [32] .

## Data Availability

The qualitative data set is not available as participants did not consent to have their data shared publicly. For data inquiries, please contact the corresponding author.
